# Genome sequence of the protist *Acanthamoeba comandoni* strain Pb30/40, morphological group I

**DOI:** 10.1128/mra.00696-25

**Published:** 2025-12-29

**Authors:** Gabriel Alexander Vignolle, Lukas Tegel, Silvia Schönthaler, Julia Walochnik, Ivan Barišić

**Affiliations:** 1 Center for Health & Bioresources, Molecular Diagnostics, AIT Austrian Institute of Technology 31189 , Vienna, Austria; 2 The Vatche and Tamar Manoukian Division of Digestive Diseases, Department of Medicine, David Geffen School of Medicine at UCLA, Los Angeles, California, USA; 3 Goodman-Luskine Microbiome Center, University of California Los Angeles, Los Angeles, California, USA; 4 Medical University of Vienna, Institute of Specific Prophylaxis and Tropical Medicine, Unit Molecular Parasitology 27271, Vienna, Austria; University of California Riverside, Riverside, California, USA

**Keywords:** eukaryota, *Acanthamoeba*

## Abstract

The free-living protist *Acanthamoeba* inhabits aquatic and soil environments, feeding on bacteria, algae, and fungi. It acts both as an opportunistic pathogen and a host or vector for pathogenic bacteria. This study presents a novel genome assembly of the *Acanthamoeba comandoni* strain Pb30/40, comprising a sequence length of 82.3 Mb.

## ANNOUNCEMENT

Organisms belonging to the eukaryotic genus *Acanthamoeba* globally constitute a rising threat, as they are the causative agents of a potentially serious eye disease, granulomatous amoebic encephalitis, and skin infections in humans and animals (1). Organisms belonging to the *Acanthamoeba* genus have been clustered based on the DNA sequence analysis of the 18S ribosomal subunit (>5% sequence dissimilarity) into 23 different genotypes (T1–T23) (2–6). This group-based classification of *Acanthamoeba* is mainly used in clinical diagnostic applications due to the availability of genotype-associated PCR primers and the simplicity of PCR-based detection. Most strains commonly acting as disease-causing agents belong to the most prevalent *A. castellanii* (T4 genotype), for which a full genome is available (7). We explored a draft genome assembly of the non-pathogenic *A. comandoni* strain Pb30/40, which was originally isolated from a greenhouse in Germany and initially classified as *A. astronyxis* and genotype T7 but now, as more genotypes have been established, shows the highest similarity to genotype T18 (8, 9).

Trophozoites were cultured at room temperature in a proteose-peptone-yeast-extract-glucose liquid medium, containing 10 g proteose peptone, 10 g yeast extract, 5 g NaCl, 5 g glucose, 0.7 g NaHPO_4_, and 0.7 g KH2PO4 per L H_2_O (10). Prior to establishing sub-cultures for DNA isolation, the amoebae were treated for 10 days with 100 µg/mL gentamycin to avoid sequencing biases due to contaminations from endosymbionts (11, 12). Amoebae were harvested by centrifugation (700 × *g*/10 min) and washed twice with phosphate-buffered saline. Subsequently, genomic DNA was isolated using the QIAmp DNA isolation kit (Qiagen, Hilden, Germany) following the manufacturer’s instructions. The whole genome shotgun sequencing was performed using the Ion Torrent PGM technology and a 318 v2 semiconductor microchip. A 400 bp DNA library was created using enzymatic fragmentation and the Ion Shear Plus Enzyme Mix II kit (Life Technologies, Carlsbad, CA, USA).

The sequencing resulted in 5,178,749 single-end reads comprising 1.62G bases with a mean sequence length of 358 bp. The *de novo* assembly was performed using SPAdes v3.15.4 (13), employing the careful parameter to reduce the number of mismatches and short indels/deletions. We used QUAST v5.2.0 (14, 15), including the eukaryotic_odb10 marker set with Benchmarking Universal Single-Copy Orthologs (BUSCO) v4 (16), for the final evaluation. The final draft genome of *A. comandoni* strain Pb30/40 consists of 43,878 scaffolds (total sequence length, 79,497,867 bp; GC content, 43.47%; N_50_, 2.1 kbp; L_50_, 11,526; mean coverage, 37×, 39.6% BUSCOS found) Comparative analysis revealed that strain Pb30/40 shows a genome similarity of 2.49% to the genotype T4 of the *A. castellanii* Neff strain published by Clarke et al. (12), based on the QUAST report. This mapping result corresponds to the dissimilarities of >30% found between the two 18S rRNA genes of the strains, when aligning them to each other. Mitochondrial reads were assembled and retained as multiple contigs in the draft genome.

This Whole Genome Shotgun project has been deposited at DDBJ/ENA/GenBank under the accession MRZZ00000000. The version described in this paper is version MRZZ02000000. The raw sequence data are available under accession number SRX2460089.
